# COMMUNITY RATES OF DEPRESSION AND DISABILITIES: FINDINGS FROM THE MS HEALTHY AGING DATA REPORTS

**DOI:** 10.1093/geroni/igae098.2683

**Published:** 2024-12-31

**Authors:** Yan-Jhu Su, Taylor Jansen, Chae Man Lee, Elizabeth Dugan, Nina Silverstein, Kina White

**Affiliations:** University of Massachusetts Boston, Boston, Massachusetts, United States; University of Massachusetts Boston, Boston, Massachusetts, United States; University of Massachusetts Boston, Boston, Massachusetts, United States; University of Massachusetts Boston, Boston, Massachusetts, United States; University of Massachusetts Boston, Needham, Massachusetts, United States; Mississippi State Department of Health, Jackson, Mississippi, United States

## Abstract

Depression in older adults is a significant public health issue, affecting all aspects of their well-being, independence, and quality of life. This cross-sectional study describes county rates of depression and daily life difficulties (ambulatory difficulty, self-care difficulty, and independent living difficulty) in Mississippi. We then explored if there was an association between county rates of depression and the disability measures in 82 counties in Mississippi. Small area estimation techniques were used to calculate rates using data from: the American Community Survey (2016-2020), the Behavioral Risk Factor Surveillance System (2013-2020), and the Centers for Medicare and Medicaid Services (2018). County rates varied: depression ranged from 6.30% to 24.9% (16.4% state average), ambulatory difficulties (7.14%-46.36%), self-care difficulties (2.53%-24.16%), and independent living difficulty (8.54%-33.0%). Multiple linear regression models after adjusting for age, gender, race, marital status, education, poverty, and physical health status, revealed that counties with higher rates of depression were significantly associated with higher rates of ambulatory difficulty and self-care difficulty (p = 0.003 and p < 0.001, respectively) but not independent living (p = 0.176). Bivariate mapping was conducted to overlay rates of depression and daily life difficulties, demonstrating counties that were high in depression and daily life difficulties in Mississippi. Identifying areas with high rates of 65+ depression and activities of daily living difficulties can help policymakers and service providers to prioritize communities most in need of interventions and services.

